# Effects of non-surgical periodontal therapy on systemic inflammation and metabolic markers in patients undergoing haemodialysis and/or peritoneal dialysis: a systematic review and meta-analysis

**DOI:** 10.1186/s12903-020-1004-1

**Published:** 2020-01-22

**Authors:** Hui Yue, Xinxin Xu, Qin Liu, Xiaozhi Li, Yiting Xiao, Bo Hu

**Affiliations:** 1 Department of Stomatology Surgery, The First Affiliated Hospital of Chongqing Medical University, Chongqing, China; 2 Stomatological Hospital of Chongqing Medical University, No. 426 Songshibei Road, Chongqing, 401147 China; 3 Chongqing Key Laboratory of Oral Diseases and Biomedical Sciences, Chongqing, China; 4 Chongqing Municipal Key Laboratory of Oral Biomedical Engineering of Higher Education, Chongqing, China; 5 School of Public Health and Management, Chongqing Medical University Chongqing, Chongqing, China

**Keywords:** End-stage renal disease, Haemodialysis, Peritoneal dialysis, Non-surgical periodontal treatment

## Abstract

**Background:**

This systematic review aimed to investigate whether non-surgical periodontal therapy (NSPT) can reduce systemic inflammatory levels and improve metabolism in patients undergoing haemodialysis (HD) and/or peritoneal dialysis (PD).

**Methods:**

Electronic databases (PubMed, EMBASE, CENTRAL, CNKI, and WFPD) were searched for randomized controlled trials (RCTs) performed through July 2019. The risk of bias within studies was assessed with the Cochrane Collaboration’s risk assessment tool. The systemic inflammatory and metabolic outcomes included the highly sensitive C-reactive protein (hs-CRP), interleukin 6 (IL-6), tumour necrosis factor-*a* (TNF-*a*), the albumin (Alb), and lipid metabolite levels. Meta-analyses (MAs) were performed to calculate the overall effect size where appropriate.

**Results:**

Five RCTs were included in this study. Compared with untreated periodontitis groups, the dialysis patients after NSPT significantly showed decreased hs-CRP levels at less than or equal to 2 months (standardized mean difference: − 1.53, 95% confidence interval − 2.95 to − 0.11). No significant difference was found in IL-6 and Alb levels following NSPT at either the 3- or 6- month follow-ups. No MAs could be performed on the TNF-*a* level and the lipid metabolic markers.

**Conclusions:**

NSPT can moderately reduce serum hs-CRP levels in HD and/or PD patients, but did not significantly change IL-6 or Alb levels. For TNF-*a* and lipid metabolism markers, no sufficient evidence supports that these levels are changed after NSPT. Additional scientific research is necessary to assess the effects of NSPT on systemic inflammation and metabolic parameters in dialysis patients.

## Background

End-stage renal disease (ESRD) is a disease in which the glomerular filtration rate continues to decline, leading to kidney failure [1, 2]. ESRD is an important public health problem with high morbidity and mortality [3]. The life-saving treatment for ESRD includes dialysis; either haemodialysis (HD) or dialysis (PD) and kidney transplantation, dialysis treatment is the most common treatment [3, 4]. Because of metabolism and immune system dysfunction, compared to non-dialysis, patients undergoing dialysis are more susceptible to suffer from elevated highly sensitive C-reactive protein (hs-CRP), interleukin 6 (IL-6), and cholesterol levels, and decreased serum albumin (Alb) levels, which contributed to an increased risk of cardiovascular disease (CVD), atherosclerosis, malnutrition and chronic periodontitis (CP). Accordingly, ESRD has been suggested as one of the risk factors of CP [5–9].

CP is a chronic infective disease mainly caused by dental plaque biofilm, characterized by the destruction of the soft and hard tissues surrounding the teeth. If not treated in time, the teeth will eventually be lost [10]. The production of local periodontal proinflammatory and inflammatory factors is related to the host’s systemic inflammatory and immunological response [11], and there may be a bidirectional relationship between ESRD and chronic periodontitis. CP has been considered a potential source of increased systemic inflammatory burden and malnutrition in dialysis patients [12–14], which may aggravate the existing metabolic, endocrine, and immune disorders in patients receiving HD and/or PD [5, 8]. In contrast, the elevated risk or severity of periodontal disease in dialysis patients may be associated with high levels of serum inflammatory biomarkers and low levels of serum Alb [15–17]. Thus, the control of systemic inflammation and metabolic markers in dialysis patients may reduce the CVD rate and atherosclerosis, improve the prognosis of ESRD and improve the pathological progression of periodontal diseases [11, 18, 19].

Periodontal therapy (PT) is a standard therapeutic modality used to control infection and inflammation in periodontal diseases [20]. PT includes surgery and non-surgical periodontal therapy (NSPT), and the latter is feasible and easily been performed by periodontal practitioners, mainly including professional oral hygiene instructions (OHI), full-mouth scaling and root planing (SRP) to remove supra/subgingival biofilm and calculus [21]. Recently, many scholars have begun to investigate and study the effects of NSPT on systemic inflammation and metabolism status of dialysis patients, but their conclusions are contradictory. Some investigators have shown that NSPT can decrease systemic inflammation and improve the nutrition and lipid metabolic status of dialysis patients [14, 22, 23]. However, other investigators have noted that NSPT does not significantly reduce systemic inflammation and improve metabolic markers in ESRD patients [24]. This inconsistency is not conducive to the clinical promotion of NSPT, the control of systemic inflammation, and the improvement of nutrition and lipid metabolism status in dialysis patients.

Hence, it was important to conduct a systematic review and meta-analysis to evaluate whether NSPT of CP can influence systemic inflammation and metabolic measures in ESRD patients undergoing HD and/or PD.

## Methods

We conducted and reported the results following the Cochrane Handbook and the Preferred Reporting Items for Systematic Reviews and Meta-Analyses (PRISMA) statement [25]. We registered it at PROSPERO (CRD42018112231).

### Focused question

Does non-surgical periodontal therapy influence systemic inflammation and metabolic markers in haemodialysis and/or peritoneal dialysis patients with chronic periodontitis?

### Search strategy

A comprehensive electronic search of PubMed, EMBASE, Cochrane Central Register of Controlled Trials (CENTRAL), China National Knowledge Infrastructure (CNKI), and Chinese Medicine Premier’s Wanfang database (WFPD) was searched from their inception to July 2019 for eligible articles. We used the following MeSH terms and free text keywords: population [“renal dialysis” OR “peritoneal dialysis” OR “haemodialysis” OR “kidney failure, chronic” OR “end-stage renal disease” OR “renal insufficiency”] AND intervention [“periodontal therapy” OR “periodontal/dental debridement” OR “non-surgical periodontal debridement” OR “dental prophylaxis/instrumentation” OR “root planing”]. We also reviewed the reference lists of identified studies and pertinent reviews for additional citations. Databases were searched to include papers published in English and Chinese. The detailed search process is illustrated in the Additional file 1. We did not contact the original authors for further information.

### Eligibility criteria

Two reviewers (HY and XXX) independently evaluated the eligible studies that met the following criteria: (1) study design: randomized controlled clinical trial (RCT); (2) the ESRD patients diagnosed with CP; further inclusion were: the patients undergoing PD and/or HD; without other sources of inflammation such as pulpal infections and active caries; no periodontal treatment in past six months; (3) intervention group of NSPT including professional OHI, full-mouth SRP, (including ultrasonic and/or hand supra/subgingival biofilm and calculus removal), SRP plus local or systemic antiseptic therapy without surgical flap procedures; (4) control group of age and gender-matched no periodontal treatment, delayed treatment or only including OHI; (5) and the follow-up times ≥4 weeks.

The exclusion criteria were reviews, studies without a comparison group, case reports, and conference abstracts.

### Screening and data extraction

Two reviewers (HY and XXX) independently performed the study screening process. First, we excluded duplicated publications and scrutinized titles and abstracts to select literature that may meet the inclusion criteria. Then, we read the full text carefully to include eligible studies further. We implemented data extraction from included studies, and then another reviewer (XZL) checked the results for accuracy. The following information was extracted: the name of first author, publication year, sample size of participants, study methods (such as study design, follow-up duration), intervention, conclusions, clinical and biochemical measures including serum inflammatory markers: hs-CRP, IL-6, TNF-*a*; metabolic markers including nutritional markers (such as the albumin, Alb) and lipid metabolic markers (including total cholesterol, TC; triglycerides, TG; high-density lipoprotein cholesterol, HDL-C; and low-density lipoprotein cholesterol, LDL-C). Any inconsistent results regarding the eligibility of studies and extraction of data occurred between the two reviewers was resolved by a conversation with a third reviewer (XZL).

### Quality assessment

Two reviewers (HY and BH) independently performed the quality assessment of the selected studies via the Cochrane Handbook for Systematic Reviews of Interventions for assessing the risk of bias. The tool included six domains: random sequence generation, allocation concealment, blinding of participants, outcome assessors, incomplete outcome data, selective reporting, and other biases. All six included issues were evaluated as low risk, high risk, or unclear. Any divergence between the two investigators was resolved by discussion with a third reviewer (QL).

### Data analysis

We (HY and QL) performed data analysis and pooled in statistical meta-analyses using Stata software (version 15.0, StataCorp, College Station, TX). In the meta-analysis, we calculated overall effect size (ES) estimates using the standardized mean difference (SMD) and the upper and lower limits of the 95% confidence intervals (CI) to assess overall efficacy from all the eligible studies. The heterogeneity was assessed by Q statistic (*P* < 0.10 indicating significant heterogeneity), and *I* squared (*I*^*2*^) statistic. An *I*^*2*^ value of more than 50% represented high heterogeneity; thus, the random effect model would be adopted. *I*^*2*^ less than 50% representing low heterogeneity, fixed-effects models were used. Statistical significance was declared if the *P*-value was < 0.05. Publication bias and sensitivity analyses would have been conducted if the included trials were at least ten according to Higgins and Green [26].

## Results

### Study selection

**Fig. 1:**
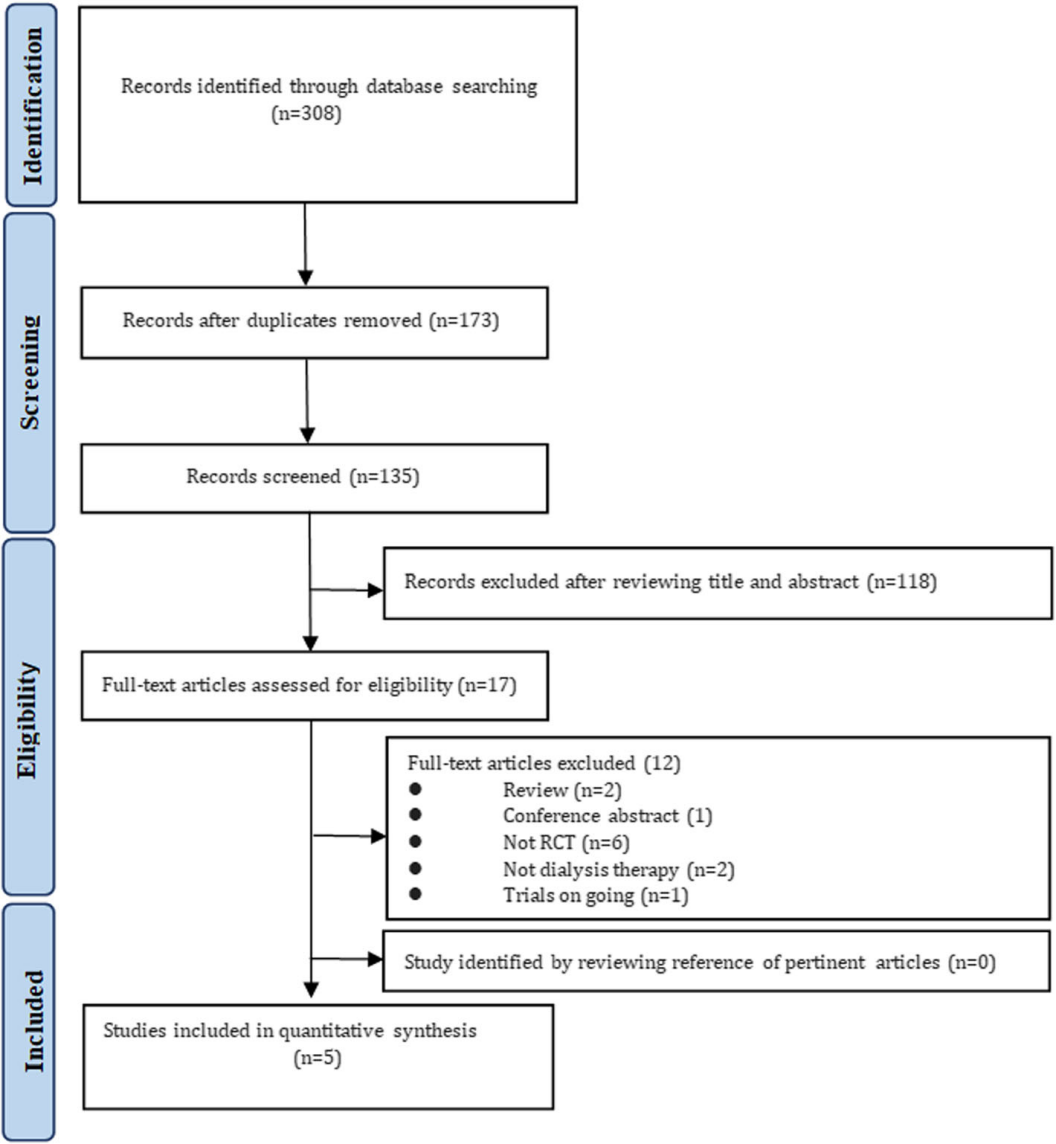
Flow diagram of the studies identified, included, and excluded

The inter-rater agreement for study screening between investigators was kappa = 0.79. In total, 308 articles were searched in the databases. After duplicates were removed, 135 articles were retained for the title and abstract identification. The full texts of 17 articles were read carefully and further assessed in strict accordance with the eligible criteria. Three RCTs were excluded; two RCTs did not include dialysis treatment [27, 28], and one was still ongoing [29]. Eventually, five RCTs were included for systematic assessment (Fig. 1). Screening the reference lists did not reveal additional suitable articles following the inclusion criteria.

### Characteristics of the included studies

Table 1 shows details of the summarized characteristics of included studies. Five included RCTs [23, 24, 30–32] were of parallel design. Fang [24], Li [32] and Ma [23] recruited HD patients, Zhang and colleagues [31] recruited PD patients, and Wehmeyer and colleagues [30] recruited HD and PD patients. The HD treatment time established by investigators of three studies was on average 4 h of HD 3 times a week [23, 24, 32]. In the study by Zhang [31] and Wehmeyer [30], the HD or PD treatment time was not mentioned. All of the included studies were at least one month follow-ups. The investigators of two studies included both a 3-month and 6-month follow-up periods [24, 30]. Four studies reported hs-CRP levels [23, 24, 31, 32], two reported IL-6 and Alb levels [24, 30], and only one reported serum TNF-*a* level and lipid metabolism markers [24]. The data of hs-CRP levels are available in 4 studies and were collected for the meta-analysis. Five studies provided approximately 353 subjects. However, in the study by Fang and colleagues, two patients were lost to 6-month follow-up owing to time conflicts in the intervention group; two patient was lost to 6-week and 3-month follow-up in the control group, respectively [24].

**Table 1:** Characteristics of Populations and Interventions in the Included trails

Study, year	No. of patients (no. of lost to follow up)	Periodontal definition	Study methods	Intervention	Outcome measures	Authors’ conclusion
Fang 2015	97 (4)	CAL ≥ 1 mm, including slight, moderate, and severe periodontitis and at least 16 teeth	RCT, 2 groups with ESRD undergoing HD, on average 4 h of HD 3 times a week; 6-week, 3- and 6-month follow-up	T: NSPT, including OHI + SRP at baseline and supragingival prophylaxis at 3 months C: OHI A single certified periodontist	After 3, 6 months, the levels of hs-CRP, IL-6 and Alb significantly decreased (*P* < 0.05); No significant difference for serum TNF-α, TC, TG, HDL-C, and LDL-C levels;	NSPT can effectively improve periodontal, systemic inflammation and nutritional status in ESRD patients
Wehmeyer 2014	25 (0)	At least 2 teeth with ≥6 mm CAL and at least 1 a site with probing depth > 5 mm	RCT, 2 groups undergoing HD and/or PD; 3- and 6-month follow-up	T: NSPT (OHI + SRP and local minocycline when all sites with PD > 5 mm at the time of SRP and at the 3 and 6 months) C: OHI One of three trained providers	No significantthe difference for serum albumin (Alb) and IL-6 levels in the intervention group at any point period (3 and 6 months)	NSPT did not produce an significant impact on serum systemic inflammation and nutritional markers
Li 2019	72 (0)	At least two sites with CAL ≥ 3 mm and probing depth ≥ 4 mm	RCT, 2 groups undergoing HD, on average 4 h of HD 3 times a week; 8-week follow-up	T: NSPT (OHI+ SRP); C: No treatment No report about who performed the therapy	Hs-CRP levels significantly decreased in the intervention group	NSPT can decrease systemic inflammation through hs-CRP
Zhang 2017	61 (0)	At least 6 sites with CAL ≥ 4 mm and at least 14 teeth	RCT, 2 groups undergoing PD; 4-week follow-up	T: NSPT (OHI+ SRP); C: No treatment No report about who performed the therapy	Hs-CRP levels significantly decreased in the intervention group	NSPT can reduce systemic inflammation through hs-CRP
Ma 2018	98 (0)	At least 2 sites with CAL>3 mm and probing depth>4 mm and at least 20 teeth	RCT, 2 groups undergoing HD, on average 4 h of HD 3 times a week; 6-week follow-up	T: NSPT (OHI+ SRP); C: No treatment No report about who performed the therapy	Hs-CRP levels significantly decreased in intervention group	NSPT can reduce systemic inflammation through hs-CRP

Abbreviations: HD, haemodialysis; PD, peritoneal dialysis; CAL, clinical attachment loss; RCT, randomized clinical trial; ESRD, end-stage renal disease; hs-CRP, high sensitive c-reactive protein; OHI, oral hygiene instructions; NSPT non-surgical periodontal therapy; SRP scaling and root planing; IL-6, interleukin 6; Alb, albumin; TNF-α, tumour necrosis factor alpha; TC, total cholesterol; TG, triglycerides; HDL-C, high-density lipoprotein cholesterol; LDL-C, low-density lipoprotein cholesterol

All studies described similar NSPT based on OHI and SRP without surgical procedures. One study combined SRP with local minocycline administered to all sites with PD > 5 mm at baseline, 3- and 6- month follow up visits [30]. Patients in the studies conducted by Zhang [31], Li [32], Ma [23], and Wehmeyer [30] did not receive prophylaxis within follow up periods. However, Fang and colleagues carried out supragingival prophylaxis at three months [24].

### Risk of bias

Table 2 presents the methodological and quality of the trials included in the review. Most of the included studies did not report random sequence generation, and none of the studies reported blinding of outcome assessment of practitioners. All considered studies were judged to exhibit a moderate-risk bias.

**Table 2:** Risk of bias assessment for included RCTs

Study	Random sequence generation	Allocation concealment	Blinding of participants and personnel	Blinding of outcome	Incomplete outcome data	Selective reporting	Other biases	Overall risk of bias
Fang 2015	low	low	low	unclear	low	low	low	moderate
Wehmeyer 2014	low	low	low	unclear	low	low	low	moderate
Li 2019	unclear	unclear	unclear	unclear	low	low	low	moderate
Zhang 2017	low	unclear	unclear	unclear	low	low	low	moderate
Ma 2018	unclear	unclear	unclear	unclear	low	low	low	moderate

Low risk of bias: six domains were assessed as “low risk”; Moderate risk of bias: one or more domains were assessed as “unclear”; High risk of bias: one or more domains were assessed as “high risk”

### Qualitative synthesis

The descriptive synthesis of the included studies is displayed in Table 1. Only one study focused on TNF-*a* and lipid metabolism [24]; thus, MAs for these markers were not possible. The levels of TNF-*a* and lipid metabolism markers showed no significant difference at most points. However, the level of HDL-C was significantly decreased at six weeks after NSPT in the HD and/or PD patients. None of the included studies reported the occurrence of adverse effects related to NSPT in the HD and/or PD patients.

### Quantitative synthesis

#### Hs-CRP

**Fig. 2:**
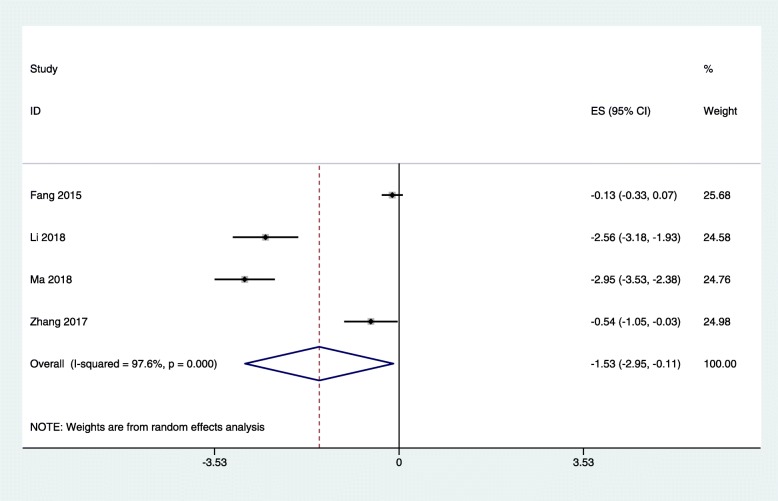
Forest plot of the difference between serum c-reactive protein intervention and control patients at less than or equal to 2 months. ES: effect size

Four studies [23, 24, 31, 32] reported the levels of hs-CRP in dialysis patients after non-surgical periodontal treatment. The results of this meta analysis showed that the level of serum hs-CRP was significantly decreased at less than or equal to 2 months (SMD: − 1.53; 95% CI: − 2.95 to − 0.11) in dialysis after NSPT, compared with untreated periodontitis patients receiving dialysis (Fig. 2), suggesting the hs-CRP levels were significantly decreased in dialysis patients with periodontitis following NSPT. The heterogeneity observed among the studies was high, so a random-effect model was used.

#### IL-6 and Alb

**Fig. 3:**
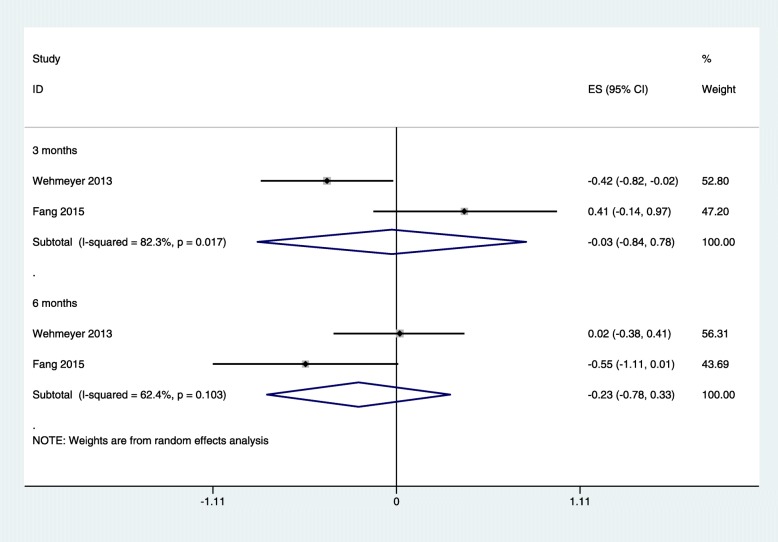
Forest plot of the difference between interleukin 6 intervention and control patients at 3 months and 6 months. ES: effect size

**Fig. 4:**
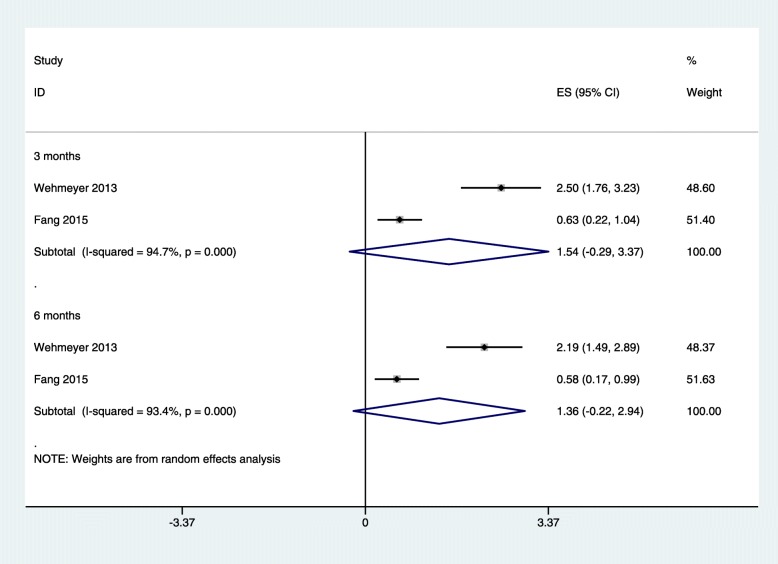
Forest plot of the difference between albumin intervention and control patients at 3 months and 6 months.ES: effect size

Two studies [24, 30] reported IL-6 and Alb levels after NSPT for HD and/or PD patients with CP. The subgroup analysis revealed that after NSPT, the levels of IL-6 in dialysis patients were decreased at 3 months (SMD: − 0.03; 95% CI: − 0.84 to 0.78) and 6 months (SMD: − 0.23; 95% CI: − 0.78 to 0.33), while there was no significant difference (Fig. 3); and the subgroup analysis revealed that the levels of Alb in the HD and/or PD patients were increased at 3 months (SMD: 1.54; 95% CI: − 0.29 to 3.37) and 6 months (SMD: 1.36; 95% CI: − 0.22 to 2.94). However, no significant difference was found (Fig. 4).

## Discussion

The present meta-analysis showed that NSPT of periodontitis, a potential source of systematic inflammation in dialysis patients, was associated with a reduction in hs-CRP levels through published RCT evidence. Local mechanical treatment of periodontitis resulted in a significantly reduced systemic inflammatory response in ESRD patients receiving dialysis. The main reason may be that NSPT can eliminate plaque, calculus, periodontal pathogens or their products and other stimulating factors in the periodontal environment. The control of periodontal infection resulted in decreased production and activity of local proinflammatory mediators and might have positive effects by reducing systemic inflammatory markers in patients receiving HD and/or PD. Emerging systematic reviews showing that the effects of NSPT-induced decrease in reducing other biochemical indicators in non-dialysis patients with periodontitis, such as glomerular filtration rate and hemoglobin Alc, support this plausible mechanism [33, 34].

To assess systematic inflammation and metabolic markers more comprehensively in dialysis patients, we also evaluated Alb and lipid metabolic markers (TC, TG, HDL-C, LDL-C). We performed a separate subgroup analysis of IL-6 and Alb levels in the 3-month and 6-month periods; however, our study showed no significant differences in IL-6 and Alb levels after NSPT in dialysis patients. Regarding contradictory results in the included studies, Fang and colleagues found that NSPT significantly increased Alb [24]. Wehmeyer and colleagues showed that the increase in Alb induced by NSPT was greater than that abserved with no treatment, while no significant differences were found. Although Wehmeyer and colleagues implemented randomization, more than 60% of the ESRD patients assigned to the treatment group also had diabetes mellitus (DM) [30]. DM is recognized as one of the most important systemic risk factors for CP [35]. DM might have detrimental effects on periodontal wound-healing ability and the response to NSPT. This may reduce the effectiveness of the treatment observed. Further well-designed studies are needed to elucidate the effects of non-surgical periodontal treatment on serum Alb and IL-6 levels.

In the present meta-analysis, we observed substantial heterogeneity in the combined SMD of serum biochemical markers after PD in ESRD patients undergoing dialysis. Heterogeneity in clinical research is inevitable. Biochemical markers are associated with common risk factors of CP and ESRD; thus, the demographics of the ESRD subjects with or without diabetes, dialysis types, dialysis time, NSPT with or without auxiliary use of antibiotics, and different follow-up times predominantly contributed to the observed heterogeneity. Considering the limited number of studies, we could not conduct subgroup analyses to account for these confounders. Thus, it should be remembered that our conclusions are based on limited studies and therefore need to be confirmed by larger, well-designed studies.

This is the first systematic review and meta-analysis that analyzed the effect of NSPT on systemic inflammation and metabolic measures in HD and/or PD patients. Four of the five included RCTs were published within the past four years. In this study, we quantitatively assessed systematic inflammation, nutritional and lipid metabolic markers and found potential increases in serum inflammation markers and biochemical measures after NSPT in dialysis patients with CP. Simultaneously, several limitations were identified in our systematic review. First, NSPT, as a traditional technique for the treatment of periodontitis, lacks sufficient original clinical trials in chronic renal diseases, especially in HD and/or PD patients with CP, so only five articles were included. Second, the heterogeneity among the selected studies associated with the presence of confounding factors, such as demographic data for the subjects with or without diabetes, dialysis types, dialysis time and different follow-up times, is a possible limitation of this study. Third, all of the included articles were evaluated as having a moderate-risk bias. Finally, we did not evaluate publication bias or perform sensitivity analyses for the small number of papers. Additionally, we qualitatively analysed the TNF-*a* level and lipid metabolic markers since the included studies provided limited information. Considering the above limitations, the effects of NSPT for the treatment of periodontitis on serum clinical and biochemical markers remain worthy of investigation.

A few studies have evaluated the effect of non-operative periodontal therapy on inflammatory markers in this population. Systematic inflammation and metabolic markers have been associated with the CVD rate and prognosis of ESRD patients. We need a large number of studies to assess the benefits of periodontal treatment in patients undergoing HD and/or PD. Additional well-designed studies are needed to provide robust evidence regarding the systematic effects of NSPT on ESRD patients undergoing HD and/or PD. A longer evaluation time may produce more comparable results of clinical and biochemical parameters in periodontitis.

## Conclusion

NSPT can moderately decrease systematic inflammation, as determined by hs-CRP in dialysis patients with CP. Routine NSPT for HD and/or PD patients with periodontitis is recommended to reduce local and systemic inflammation. However, only a few studies regarding the levels of IL-6, and TNF-*a*, Alb, and lipid metabolic markers have been conducted, and no sufficient evidence supports the change in these markers after NSPT in ESRD patients. Additional well-designed studies with longer evaluation periods are required to explore the effects of NSPT on clinical and biochemical parameters in ESRD patients.

## Additional file

**Additional file 1.** The detailed search strategies in PubMed, EMBASE, CENTRAL, CNKI, WFPD databases.

## Data Availability

The data supporting the findings are available in the databases PubMed, CNKI, and WFPD.

## Abbreviations

Alb: Albumin
CI: Confidence interval
CP: Chronic periodontitis
CVD: Cardiovascular disease
ESRD: End-stage renal disease
HD: Haemodialysis
HDL-C: High-density lipoprotein cholesterol
hs-CRP: Highly sensitive C-reactive protein
IL-6: Interleukin 6
LDL-C: Low-density lipoprotein cholesterol
MAs: Meta-analyses
OHI: Oral hygiene instructions
PD: Peritoneal dialysis
PRISMA: Transparent Reporting of Systematic Reviews and Meta-analyses
PROSPERO: Prospective Register of Systematic Review
RCT: Randomized controlled trial
SMD: Standardized mean difference
SRP: Scaling and root planning
TC: Total cholesterol
TG: Triglycerides
TNF-*a*: Tumour necrosis factor-*a*
