# Supplementary material for: Effects of non-surgical periodontal therapy on systemic inflammation and metabolic markers in patients undergoing haemodialysis and/or peritoneal dialysis: a systematic review and meta-analysis
Source: BMC Oral Health. 2020 Jan 22;20:18. doi: 10.1186/s12903-020-1004-1 (PMC6977292; doi:10.1186/s12903-020-1004-1)
Supplement: Supplementary file 1 — Additional file 1. The detailed search strategies in PubMed, EMBASE, CENTRAL, CNKI, WFPD databases. [file 12903_2020_1004_MOESM1_ESM.docx]

**PubMed**

((((((((((((((((Renal Dialysis[Title/Abstract]) OR Dialyses, Renal[Title/Abstract]) OR Renal Dialyses[Title/Abstract]) OR Dialysis, Renal[Title/Abstract]) OR Hemodialysis[Title/Abstract]) OR Hemodialyses[Title/Abstract]) OR Dialysis, Extracorporeal[Title/Abstract]) OR Dialyses, Extracorporeal[Title/Abstract]) OR Extracorporeal Dialyses[Title/Abstract]) OR Extracorporeal Dialysis[Title/Abstract])) OR "Renal Dialysis"[Mesh]))) OR ((((((Dialyses, Peritoneal[Title/Abstract]) OR Dialysis, Peritoneal[Title/Abstract]) OR Peritoneal Dialyses[Title/Abstract]) OR Peritoneal Dialysis[Title/Abstract])) OR "Peritoneal Dialysis"[Mesh])) OR ((((((((((((((((((End-Stage Kidney Disease[Title/Abstract]) OR Disease, End-Stage Kidney[Title/Abstract]) OR End Stage Kidney Disease[Title/Abstract]) OR Kidney Disease, End-Stage[Title/Abstract]) OR Chronic Kidney Failure[Title/Abstract]) OR End-Stage Renal Disease[Title/Abstract]) OR Disease, End-Stage Renal[Title/Abstract]) OR End Stage Renal Disease[Title/Abstract]) OR Renal Disease, End-Stage[Title/Abstract]) OR Renal Disease, End Stage[Title/Abstract]) OR Renal Failure, End-Stage[Title/Abstract]) OR End-Stage Renal Failure[Title/Abstract]) OR Renal Failure, End Stage[Title/Abstract]) OR Renal Failure, Chronic[Title/Abstract]) OR Chronic Renal Failure[Title/Abstract]) OR ESRD[Title/Abstract])) OR "Kidney Failure, Chronic"[Mesh])) AND (((((((((((((((((Debridement, Periodontal[Title/Abstract]) OR Debridements, Periodontal[Title/Abstract]) OR Nonsurgical Periodontal Debridement[Title/Abstract]) OR Periodontal Debridements[Title/Abstract]) OR Debridement, Nonsurgical Periodontal[Title/Abstract]) OR Debridements, Nonsurgical Periodontal[Title/Abstract]) OR Nonsurgical Periodontal Debridements[Title/Abstract]) OR Periodontal Debridement, Nonsurgical[Title/Abstract]) OR Oral Hygiene Instruction*[Title/Abstract]) OR OHI*[Title/Abstract]) OR Dental Scaling/instrumentation[Title/Abstract]) OR Ultrasonic Therapy/instrumentation[MH]) OR Dental Prophylaxis/instrumentation[MH]) OR Periodontal debridement[Title/Abstract]) OR Root planing[Title/Abstract]) OR Periodontal therapy[Title/Abstract]) OR Periodontal treatment[Title/Abstract])

**EMBASE**

| No. | Query | Results |
| --- | --- | --- |
| #11 | #7 AND #10 | 117 |
| #10 | #8 OR #9 | 22022 |
| #9 | 'debridement, periodontal':ab,ti OR 'debridements, periodontal':ab,ti OR 'periodontal debridements':ab,ti OR 'nonsurgical periodontal debridement':ab,ti OR 'debridement, nonsurgical periodontal':ab,ti OR 'debridements, nonsurgical periodontal':ab,ti OR 'nonsurgical periodontal debridements':ab,ti OR 'periodontal debridement, nonsurgical':ab,ti OR 'periodontal debridements, nonsurgical':ab,ti OR 'periodontal pocket debridement':ab,ti OR 'debridement, periodontal pocket':ab,ti OR 'debridements, periodontal pocket':ab,ti OR 'periodontal pocket debridements':ab,ti OR 'oral hygiene instruction*':ab,ti OR 'ohi*':ab,ti OR 'dental scaling/instrumentation':ab,ti OR 'ultrasonic therapy/instrumentation':ab,ti OR 'dental prophylaxis/instrumentation':ab,ti OR 'periodontal debridement':ab,ti OR 'root planing':ab,ti OR 'periodontal therapy':ab,ti OR 'periodontal treatment':ab,ti | 21917 |
| #8 | 'dental debridement'/exp | 172 |
| #7 | #1 OR #2 OR #3 OR #4 OR #5 OR #6 | 224708 |
| #6 | 'end-stage kidney disease':ab,ti OR 'disease, end-stage kidney':ab,ti OR 'end stage kidney disease':ab,ti OR 'kidney disease, end-stage':ab,ti OR 'chronic kidney failure':ab,ti OR 'end-stage renal disease':ab,ti OR 'disease, end-stage renal':ab,ti OR 'end stage renal disease':ab,ti OR 'renal disease, end-stage':ab,ti OR 'renal disease, end stage':ab,ti OR 'renal failure, end-stage':ab,ti OR 'end-stage renal failure':ab,ti OR 'renal failure, end stage':ab,ti OR 'renal failure, chronic':ab,ti OR 'chronic renal failure':ab,ti OR 'esrd':ab,ti | 89840 |
| #5 | 'end stage renal disease'/exp | 26669 |
| #4 | 'dialysis, peritoneal':ab,ti OR 'peritoneal dialyses':ab,ti OR 'peritoneal dialysis':ab,ti | 31560 |
| #3 | 'peritoneal dialysis'/exp | 41161 |
| #2 | 'renal dialysis':ab,ti OR 'dialyses, renal':ab,ti OR 'renal dialyses':ab,ti OR 'dialysis, renal':ab,ti OR 'hemodialysis':ab,ti OR 'hemodialyses':ab,ti OR 'dialysis, extracorporeal':ab,ti OR 'dialyses, extracorporeal':ab,ti OR 'extracorporeal dialyses':ab,ti OR 'extracorporeal dialysis':ab,ti | 85254 |
| #1 | 'hemodialysis'/exp | 104806 |

**The Cochrane Library (CENTRAL)**

#1 MeSH descriptor: [Renal Dialysis] explode all trees

#2 MeSH descriptor: [Peritoneal Dialysis] explode all trees

#3 MeSH descriptor: [Kidney Failure, Chronic] explode all trees

#4 (Renal Dialysis):ti,ab,kw OR (Dialyses, Renal):ti,ab,kw OR (Renal Dialyses):ti,ab,kw OR (Dialysis, Renal):ti,ab,kw OR (Hemodialysis):ti,ab,kw OR (Hemodialyses):ti,ab,kw OR (Dialysis, Extracorporeal):ti,ab,kw OR (Dialyses, Extracorporeal):ti,ab,kw OR (Extracorporeal Dialyses):ti,ab,kw OR (Extracorporeal Dialysis):ti,ab,kw OR (Dialysis, Peritoneal):ti,ab,kw OR (Peritoneal Dialyses):ti,ab,kw OR (Peritoneal Dialysis):ti,ab,kw OR (End-Stage Kidney Disease):ti,ab,kw OR (Disease, End-Stage Kidney):ti,ab,kw OR (End Stage Kidney Disease):ti,ab,kw OR (Kidney Disease, End-Stage):ti,ab,kw OR (Chronic Kidney Failure):ti,ab,kw OR (End-Stage Renal Disease):ti,ab,kw OR (Disease, End-Stage Renal):ti,ab,kw OR (End Stage Renal Disease):ti,ab,kw OR (Renal Disease, End-Stage):ti,ab,kw OR (Renal Disease, End Stage):ti,ab,kw OR (Renal Failure, End-Stage):ti,ab,kw OR (End-Stage Renal Failure):ti,ab,kw OR (Renal Failure, End Stage):ti,ab,kw OR (Renal Failure, Chronic):ti,ab,kw OR (Chronic Renal Failure):ti,ab,kw OR (ESRD):ti,ab,kw

#5 #1 OR #2 OR #3 OR #4

#6 (Debridement, Periodontal):ti,ab,kw OR (Debridements, Periodontal):ti,ab,kw OR (Periodontal Debridements):ti,ab,kw OR (Nonsurgical Periodontal Debridement):ti,ab,kw OR (Debridement, Nonsurgical Periodontal):ti,ab,kw OR (Debridements, Nonsurgical Periodontal):ti,ab,kw OR (Nonsurgical Periodontal Debridements):ti,ab,kw OR (Periodontal Debridement, Nonsurgical):ti,ab,kw OR (Periodontal Debridements, Nonsurgical):ti,ab,kw OR (Periodontal Pocket Debridement):ti,ab,kw OR (Debridement, Periodontal Pocket):ti,ab,kw OR (Debridements, Periodontal Pocket):ti,ab,kw OR (Periodontal Pocket Debridements):ti,ab,kw OR (Oral Hygiene Instruction*):ti,ab,kw OR (OHI*):ti,ab,kw OR (Dental Scaling):ti,ab,kw OR (Ultrasonic Therapy):ti,ab,kw OR (Dental Prophylaxis):ti,ab,kw OR (Periodontal Debridement):ti,ab,kw OR (Root Planing):ti,ab,kw OR (periodontal therapy):ti,ab,kw OR (periodontal treatment):ti,ab,kw

#7 #5 AND #6

**CNKI**

("肾透析"[常用字段] OR "体外透析"[常用字段] OR "血液透析"[常用字段] OR "肾透析"[主题词]) OR ("肾功能不全"[常用字段] OR "肾功能衰竭"[常用字段] OR "肾机能不全"[常用字段] OR "肾衰竭"[常用字段] OR "肾功能不全"[主题词]) OR ("腹膜透析"[常用字段] OR "腹膜透析"[主题词]) AND ("牙周清创术"[常用字段] OR "非手术牙周清创术"[常用字段] OR "牙周清创术"[主题词]) AND ("随机对照试验"[常用字段] OR "随机对照试验"[主题词])

**WFPD**

主题:(肾病+透析+血液透析+腹膜透析) * 主题:(牙周) * 主题:(随机) * Date:-2019 DBID:WF_QK
